# Swap and stop – Kinetochores play error correction with microtubules: Mechanisms of kinetochore–microtubule error correction

**DOI:** 10.1002/bies.202100246

**Published:** 2022-03-08

**Authors:** Harinath Doodhi, Tomoyuki U. Tanaka

**Affiliations:** ^1^ Centre for Gene Regulation and Expression School of Life Sciences University of Dundee Dundee UK

**Keywords:** aurora B kinase, chromosome biorientation, chromosome segregation, Dam1 complex, error correction, INCENP, kinetochore–microtubule interactions, Ndc80 complex

## Abstract

Correct chromosome segregation in mitosis relies on chromosome biorientation, in which sister kinetochores attach to microtubules from opposite spindle poles prior to segregation. To establish biorientation, aberrant kinetochore–microtubule interactions must be resolved through the error correction process. During error correction, kinetochore–microtubule interactions are exchanged (swapped) if aberrant, but the exchange must stop when biorientation is established. In this article, we discuss recent findings in budding yeast, which have revealed fundamental molecular mechanisms promoting this “swap and stop” process for error correction. Where relevant, we also compare the findings in budding yeast with mechanisms in higher eukaryotes. Evidence suggests that Aurora B kinase differentially regulates kinetochore attachments to the microtubule end and its lateral side and switches relative strength of the two kinetochore–microtubule attachment modes, which drives the exchange of kinetochore–microtubule interactions to resolve aberrant interactions. However, Aurora B kinase, recruited to centromeres and inner kinetochores, cannot reach its targets at kinetochore–microtubule interface when tension causes kinetochore stretching, which stops the kinetochore–microtubule exchange once biorientation is established.

## Graphical abstract

Chromosome segregation relies on chromosome biorientation, where sister kinetochores attach to microtubules from opposite spindle poles. To establish biorientation, aberrant kinetochore–microtubule interactions must be resolved through the error correction process. How kinetochore–microtubule interactions are exchanged during error correction and how this stops when bi‐orientation is established are discussed.

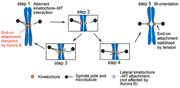

**Abbreviations**
CPC: chromosomal passenger complex
MT: microtubule
SPB: spindle pole body

## INTRODUCTION

Accurate chromosome segregation to daughter cells during mitosis is crucial for genetic integrity. This relies on the formation of correct interactions between sister chromatids and microtubules (MTs) from the opposite spindle poles. MTs are dynamic hollow tube‐like structures that exhibit phases of growth and shrinkage by addition and removal of tubulins from their plus ends.^[^ ^1^, ^2^ ^]^ Dynamic microtubules extend from spindle poles, which are organized by spindle poles bodies (SPBs) in yeast and centrosomes in metazoan cells. The interactions between chromosomes and dynamic MTs are facilitated by the kinetochore, a macromolecular complex that assembles at centromeric regions of chromosomes.^[^ ^3^ ^]^ In budding yeast *Saccharomyces cerevisiae*, kinetochores attach to the MTs extending from an SPB for most part of the cell cycle. However, they transiently detach from MTs when kinetochores disassemble during centromere replication, and re‐establish attachment after reassembly of kinetochores.^[^ ^4^ ^]^

**FIGURE 1:**
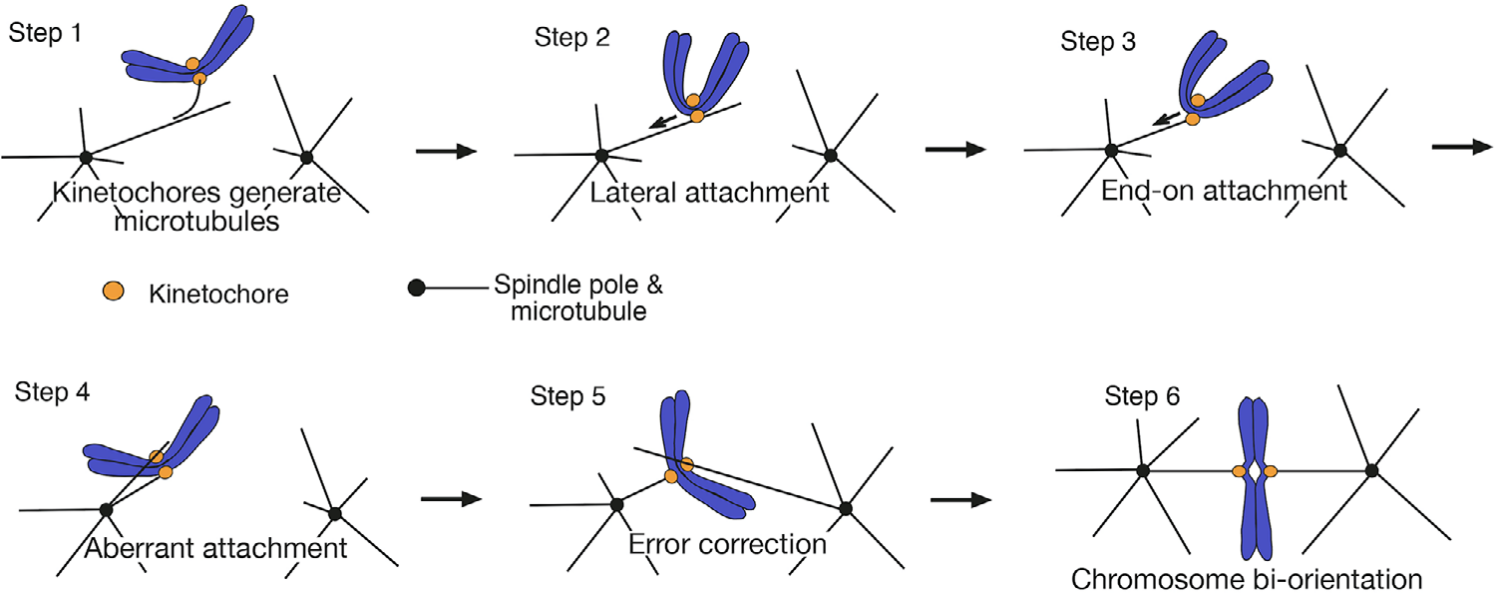
The steps for establishing correct kinetochore–MT interactions in early mitosis.^[^ ^5^ ^]^ Each step is explained in text

Kinetochore‐microtubule interaction occurs in a step wise manner^[^ ^5^ ^]^ (Figure 1). Kinetochores first attach to the lateral side of a MT (lateral attachment) extending from a spindle pole (spindle‐pole MT). This step is evolutionarily conserved from budding yeast to vertebrate cells^[^ ^6^, ^7^ ^]^ (Figure 1, step 2). The lateral attachment is often facilitated by a short MT, generated at the kinetochore, which subsequently interacts with a spindle‐pole MT.^[^ ^8^, ^9^, ^10^, ^11^ ^]^ The kinetochore‐derived MT is short‐lived and depolymerizes once the kinetochore is loaded on a spindle‐pole MT in budding yeast^[^ ^8^ ^]^ while it remains for longer as a part of the spindle in fly cells^[^ ^9^ ^]^ (Figure 1, step 1). Subsequently, the laterally attached kinetochore is transported along a spindle‐pole MT toward a spindle pole – this transport is driven by Kar3 (kinesin‐14) motor in budding yeast and by dynein motor in animal cells^[^ ^6^, ^12^, ^13^ ^]^ (step 2). As the spindle‐pole MT shrinks and its plus end catches up with the kinetochore, the kinetochore is tethered at the MT end (end‐on attachment), thus lateral attachment is converted to end‐on attachment^[^ ^14^, ^15^ ^]^ (step 3). Then, the kinetochore is pulled toward a spindle pole, as the end‐on attached MT depolymerizes. If sister kinetochores subsequently attach to MTs extending from the same pole (called aberrant or erroneous attachments; step 4), such attachment must be resolved through the process called error correction^[^ ^5^, ^16^, ^17^ ^]^ (step 5). Once sister kinetochores attach to MTs extending form opposite poles (chromosome biorientation), tension is applied across sister kinetochore and kinetochore–MT interactions are stabilized^[^ ^5^, ^16^, ^17^ ^]^ (step 6).

Chromosome biorientation is essential for correct chromosome segregation and therefore must be established before anaphase onset (Figure 1, step 6). Error correction is fundamentally important to ensure chromosome biorientation and correct chromosome segregation (steps 4, 5). The earlier steps (steps 1–3) also contribute to efficient kinetochore–MT interactions and correct chromosome segregation. Several such contributions have been reported: First, the MT lateral side provides a larger surface area for the kinetochore interaction than the MT end, thus making the initial kinetochore interaction more efficient^[^ ^6^, ^7^ ^]^ (step 2). Second, removal of kinetochore‐derived MTs suggested that they are required for more efficient kinetochore interaction with the lateral surface of spindle‐pole MTs (step 1).^[^ ^11^ ^]^ Third, two or more kinetochores on different chromosomes sometimes attach to the lateral side of the same MT in budding yeast. However, only one kinetochore can form the end‐on attachment, causing the other kinetochore(s) to detach from the MT.^[^ ^18^ ^]^ The kinetochore transport along the MT lateral side (step 2) makes this detachment happen closer to a spindle pole. As the MT density is higher around a spindle pole, this means that the detached kinetochore subsequently interacts with another MT more efficiently. Thus, kinetochore transport during the lateral attachment (step 2) indirectly facilitates the kinetochore interaction with another MT in case the original interaction is lost.^[^ ^18^ ^]^ Fourth, in both yeast and metazoan cells, the end‐on attachment is load‐bearing, that is, withstand a larger force,^[^ ^19^, ^20^ ^]^ and therefore more suitable for maintenance of biorientation. Thus, the lateral attachment must be converted to the end‐on attachment before or when biorientation is established (step 3).

**FIGURE 2:**
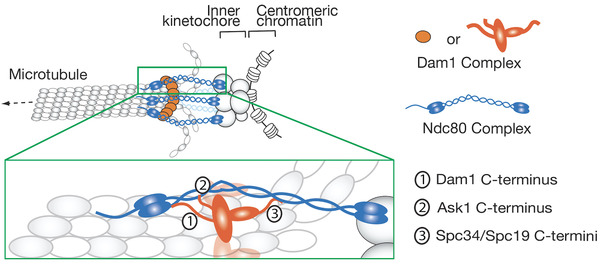
Diagram shows a model about interactions between Dam1C and Ndc80C in the end‐on kinetochore–MT attachment in budding yeast.^[^ ^27^, ^28^ ^]^ Three flexible regions of the Dam1C (Dam1C‐terminus, Ask1 C‐terminus and Spc34/Spc19 C‐termini) interact with different regions of the Ndc80C

Kinetochore is a large complex consisting of dozens of proteins, which are broadly categorized into outer and inner kinetochore components. While the inner kinetochore components bind (or localize closely to) chromosome DNA, the outer kinetochore components form the interface between the kinetochore and a MT. Among outer kinetochore components, the Ndc80 complex (Ndc80C) and the Dam1 complex (Dam1C) directly interact with MTs and play major roles in making the kinetochore–MT interface in budding yeast^[^ ^20^, ^21^, ^22^ ^]^ (Figure 2). The Ndc80C is an integral kinetochore component consisting of four proteins (Ndc80, Nuf2, Spc24, and Spc25) and essential for both lateral and end‐on attachment.^[^ ^6^ ^]^ By contrast, the Dam1C (consisting of ten different proteins including Dam1) is not present at the kinetochore during the lateral attachment but accumulates at the dynamic MT plus end.^[^ ^4^, ^15^ ^]^ When the lateral attachment is converted to the end‐on attachment (Figure 1, step 3), Ndc80Cs at the kinetochore interact with Dam1Cs at the MT end, stabilizing end‐on kinetochore–MT interface.^[^ ^23^, ^24^, ^25^, ^26^ ^]^ This interface subsequently withstands tension across sister kinetochores when biorientation is established. Chemical crosslink data suggest that three unstructured regions of Dam1C components (Dam1, Ask1, Spc34/Spc19) interact with different regions of the Ndc80C^[^ ^27^, ^28^ ^]^ – these interactions might stabilize the end‐on attachment (Figure 2).

As aberrant attachment is often formed in early mitosis (Figure 1, step 4), error correction is essential to ensuring chromosome biorientation (step 5, 6), and Aurora B kinase (called Ipl1 in budding yeast) plays a central role in promoting error correction.^[^ ^5^, ^29^, ^30^, ^31^, ^32^, ^33^ ^]^ Aurora B forms the chromosomal passenger complex (CPC) together with INCENP, Survivin and Borealin (called Sli15, Bir1, and Nbl1, respectively, in budding yeast).^[^ ^34^ ^]^ The CPC localizes at the centromere and inner kinetochore.^[^ ^35^, ^36^, ^37^ ^]^ In the absence of tension across sister kinetochores, Aurora B phosphorylates the Dam1C and Ndc80C, weakening and disrupting kinetochore–MT interaction, in budding yeast.^[^ ^38^, ^39^, ^40^ ^]^ Phosphorylation of the Dam1C by Aurora B is essential for error correction while Ndc80C phosphorylation (at Ndc80 N‐terminus) modestly contributes to error correction. Intriguingly, the three unstructured regions of the Dam1C components, which are involved in interactions with Ndc80C (Figure 2), are primary targets of phosphorylation by Aurora B.^[^ ^28^, ^38^ ^]^ It is suggested that phosphorylation of these regions by Aurora B disrupts the interaction between Ndc80C and Dam1C,^[^ ^26^, ^28^, ^41^ ^]^ leading to loss of end‐on attachment, when tension cannot be applied due to aberrant kinetochore–MT interactions – or, more specifically, due to syntelic attachment where sister kinetochores interact with MTs extending from the same spindle pole (Figure 1, step 4).

Loss of end‐on attachment is followed by formation of new kinetochore–MT interaction (Figure 1, step 5). If new interaction leads to syntelic attachment (step 4), it should be resolved again by the action of Aurora B. However, if new interaction leads to biorientation, tension is applied across sister kinetochores (step 6), which stabilizes kinetochore–MT interaction.^[^ ^17^, ^42^ ^]^ While several regulators for biorientation (e.g., Aurora B) have been identified or proposed, it is not completely understood how erroneous kinetochore–MT interaction is replaced with new interaction, that is, how kinetochore–MT interactions are exchanged (swapped), during error correction. It is also not completely clear how tension stops this exchange and stabilizes kinetochore–MT interactions. In this article, we discuss these two topics, that is, mechanisms of “swap and stop” of kinetochore–MT interactions during error correction leading to biorientation. We focus on recent findings in budding yeast *S. cerevisiae*, and also compare them with findings in higher eukaryotes where relevant. In budding yeast, only one MT attaches to a single kinetochore in metaphase,^[^ ^43^ ^]^ which should make mechanisms of error correction relatively simpler. Please note that it is not our intention to give an extensive review about chromosome biorientation across various species including animal cells.

## HOW KINETOCHORE–MT INTERACTIONS ARE SWAPPED FOR ERROR CORRECTION

It is well established that erroneous kinetochore–MT interactions are resolved by the action of Aurora B kinase (see INTRODUCTION). For this, erroneous interactions are weakened and disrupted, followed by formation of new kinetochore–MT interactions. New interactions should be frequently formed for efficient error correction. However, if Aurora B disrupts erroneous kinetochore–MT interaction, it would be straightforward to assume that Aurora B equally discourages new kinetochore–MT interactions. Alternatively, new interactions may be formed, if kinetochore phosphorylation by Aurora B is rapidly removed after erroneous kinetochore–MT interaction is disrupted but before new interaction is formed – however, such mechanism is not found or suggested. Then, how does Aurora B still allow formation of new kinetochore–MT interactions?

### Aurora B differentially regulates lateral and end‐on attachment

A clue to this question was obtained when it was investigated how the action of Aurora B affects the kinetochore interaction with the side and plus end of a MT (lateral and end‐on attachment, respectively) in early mitosis of budding yeast. To visualize clearly the lateral and end‐on attachment, an engineered assay was used, in which a chosen fluorescence‐marked centromere was inactivated and displaced from the spindle.^[^ ^6^, ^44^ ^]^ Subsequently, the centromere was reactivated to allow observation of its interaction with a long MT extending from a spindle pole. On a long MT, the lateral and end‐on attachments were clearly distinguished. Intriguingly, Dam1 phospho‐mimic mutants at four Aurora B phosphorylation sites (at the Dam1 C‐terminus; Dam1‐4D) weakened end‐on attachment, but did not affect the lateral attachment.^[^ ^26^ ^]^ Moreover, Ndc80 phospho‐mimic mutants at seven Aurora B phosphorylation sites (at the N‐terminus; Ndc80‐7D) modestly weakened the end‐on attachment, but did not affect the lateral attachment. These results suggest that the activity of Aurora B kinase differentially regulates the end‐on and lateral attachment.^[^ ^26^ ^]^ Finally, without using phosphomimic mutants, experiments with minichromosomes suggested that a physiological Aurora B activity is sufficient for such differential regulation of end‐on and lateral attachments.^[^ ^26^ ^]^

**FIGURE 3:**
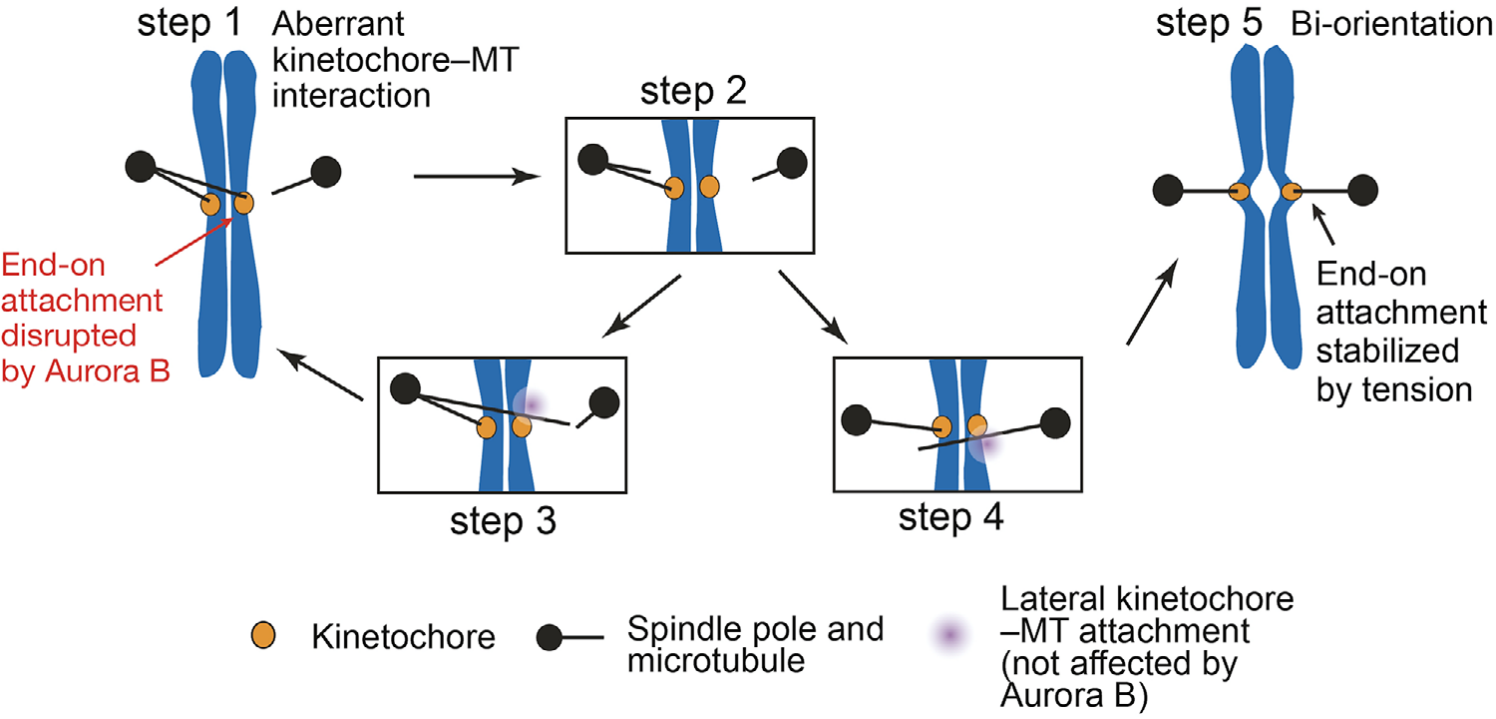
Diagram shows a model about the process of error correction, in which aberrant kinetochore–MT interaction is resolved to establish biorientation.^[^ ^26^ ^]^ Each step is explained in text. Steps 1–4 represent “Swap’ and step 5 represents “Stop” in regulation of kinetochore‐MT interactions by Aurora B. This figure was taken from Doodhi et al^[^ ^41^ ^]^

Based on these results, we have proposed the following model in which this differential regulation drives the exchange of kinetochore–MT interactions during error correction^[^ ^26^ ^]^ (Figure 3). An end‐on attachment (making erroneous kinetochore–MT interaction) is disrupted mainly due to phosphorylation of Dam1 by Aurora B (Figure 3, steps 1 and 2) and subsequently replaced by the lateral attachment to another MT, which is not inhibited by Aurora B‐dependent Dam1 phosphorylation (steps 3 and 4). The lateral attachment is then converted to end‐on attachment and, if this results in aberrant attachment, it must be resolved again by the action of Aurora B (step 1). However, if biorientation is formed, then tension is applied across sister kinetochores, stabilizing end‐on attachment (step 5).

### Aurora B switches the relative strength of lateral and end‐on attachment

More recently, the above model was tested further by reconstituting kinetochore–MT interface in budding yeast in vitro.^[^ ^41^ ^]^ For this, Ndc80Cs were attached to nanobeads (diameter ≈100 nm) and their behaviors were analyzed on Dam1C‐loaded dynamic MTs in vitro. In this system, Dam1Cs showed accumulation at the end of a depolymerising MT in vitro,^[^ ^41^ ^]^ as they do in vivo, that is, in cells.^[^ ^4^, ^15^ ^]^ The Ndc80C‐nanobeads were attached to the lateral side of a MT in vitro and then tethered at the end of the MT while it depolymerized,^[^ ^41^ ^]^ like the authentic kinetochores in vivo.^[^ ^4^, ^15^ ^]^ Dam1C was required to stabilize tethering Ndc80C‐nanobeads at the MT end.

**FIGURE 4:**
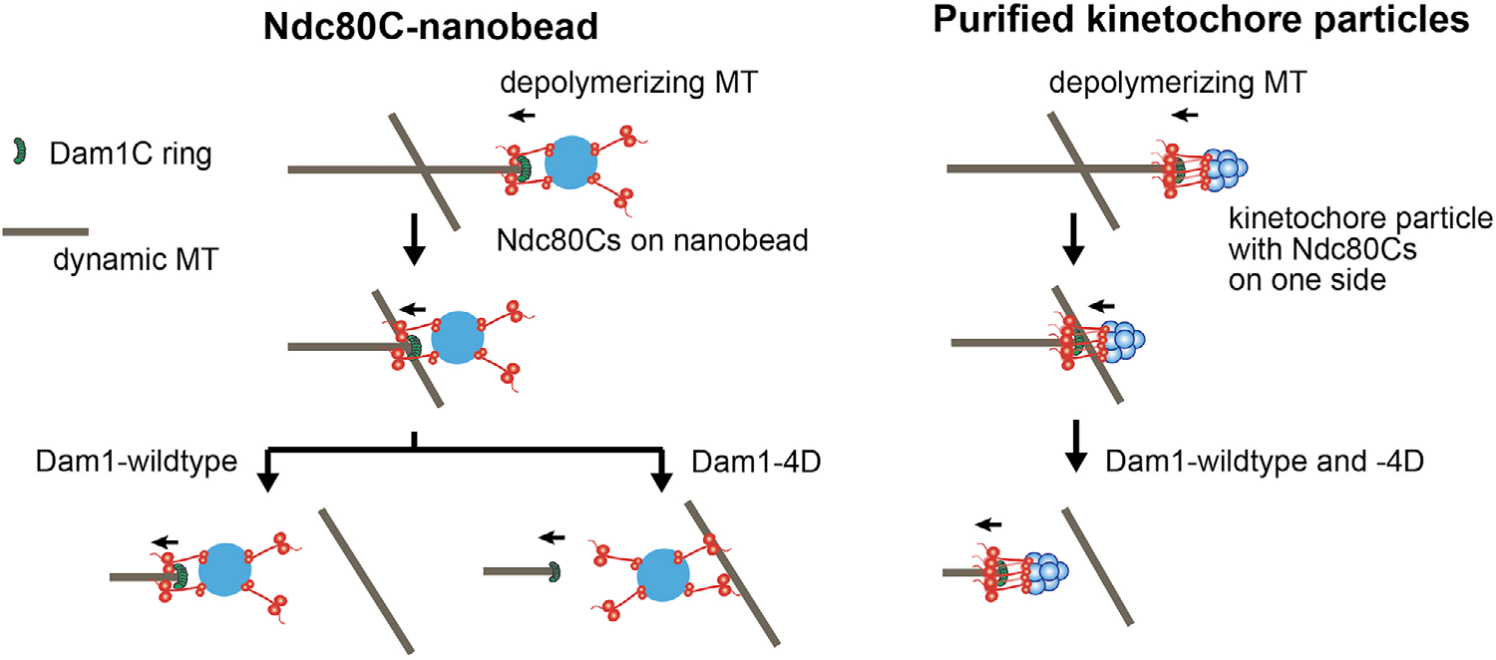
Transfer (or no transfer) of an Ndc80C‐attached nanobead (left) and a kinetochore particle (right) from the end of a depolymerizing MT to the side of another MT in vitro in the presence of Dam1‐wildtype (WT) or ‐4D (Dam1 phospho‐mimic mutants at Aurora B phosphorylation sites).^[^ ^41^ ^]^ Dam1‐WT represents nonphosphorylated Dam1 as Aurora B kinase is not present in the systems. This figure was taken from Doodhi et al.^[^ ^41^ ^]^ after modification

Using this assay system, we observed situations where two MTs crossed each other, one of which had an end‐on attachment to an Ndc80C‐nanobead during MT depolymerization^[^ ^41^ ^]^ (Figure 4, left). With wild‐type Dam1, the end‐on attachment continued and the Ndc80C‐nanobead passed across the other MT. By contrast, with Dam1 phosphomimic mutants at Aurora B phosphorylation sites (Dam1‐4D), the Ndc80C‐nanobead was often transferred from the end of the original MT to the lateral side of the other MT as the depolymerizing MT crossed it (Figure 4, left). In addition, the behaviors of Ndc80C‐nanobeads were investigated with Ndc80 wild‐type and Ndc80 phosphomimic mutants (Ndc80‐7D), but their behaviors were very similar in these two conditions.^[^ ^41^ ^]^ Whether a Ndc80C‐nanobead is transferred or not from the MT end to the lateral side of another MT would reflect the relative strength of end‐on and lateral attachments. Thus, these results suggest that Dam1 C‐terminus phosphorylation by Aurora B kinase plays a crucial role in changing the relative strength of the end‐on and lateral attachments, as follows: the end‐on attachment is stronger in the absence of Dam1 phosphorylation, but it often becomes weaker than the lateral attachment when Dam1 is phosphorylated by Aurora B. The change in the relative strength of the two modes of kinetochore–MT attachments likely promotes the exchange of kinetochore–MT interactions, that is, from end‐on attachment on one MT to the lateral attachment on another MT (Figure 3). Because the in vitro system includes only Ndc80C and Dam1C of the kinetochore, these two components sufficiently account for the differential regulation of end‐on and lateral attachment by Aurora B during error correction.

How does Dam1 C‐terminus phosphorylation by Aurora B switch relative strength of end‐on and lateral attachment? The Dam1Cs accumulate at the MT end and this Dam1C fraction supports the end‐on attachment by interacting with Ndc80Cs.^[^ ^15^, ^23^, ^24^, ^25^, ^26^, ^45^ ^]^ Dam1 C‐terminus phosphorylation would weaken the interaction with Ndc80C, thus leading to disruption of the end‐on attachment. Indeed, using the above in vitro system, it was shown that Dam1C–Ndc80C interaction was disrupted, when an Ndc80C‐nanobead lost end‐on attachment and was transferred to the lateral side of another MT.^[^ ^41^ ^]^ The Dam1C can also localize along a MT,^[^ ^15^, ^46^, ^47^ ^]^ but this fraction does not seem to be important for the lateral attachment or for Aurora B‐dependent regulation of kinetochore–MT interactions. Indeed, the Dam1 C‐terminus is not required for the lateral attachment.^[^ ^26^ ^]^ Thus, when Dam1 C‐terminus is phosphorylated by Aurora B, the end‐on attachment is weakened but the strength of lateral attachment is unchanged, resulting in the switch of relative strength of the two kinetochore–MT interaction modes.

### The context and timing for disruption of end‐on attachment during error correction

When Ndc80C‐nanobeads were transferred from the MT end to the side of another MT in the presence of Dam1 phospho‐mimic mutants (Dam1‐4D), the end‐on attachment was not lost until the lateral attachment was formed, that is, Ndc80C‐nanobeads were always attached to one MT or transiently to two MTs during the transfer^[^ ^41^ ^]^ (Figure 4, left). In other words, they were transferred “directly” from a MT to another. It is possible that the direct transfer reflects physiological behavior of native kinetochores during error correction. In fact, it was previously implied that erroneous MT attachments were not released from the kinetochore until a new attachment is formed in grasshopper spermatocytes.^[^ ^17^, ^48^ ^]^ To investigate the MT exchange at native kinetochores, native kinetochore particles were purified from budding yeast^[^ ^49^ ^]^ and it was investigated whether they were directly transferred between MTs in vitro as were the Ndc80C‐nanobeads. Interestingly, the kinetochore particles had end‐on attachment maintained when passing across the side of another MT in the presence of either Dam1 wild‐type or ‐4D^[^ ^41^ ^]^ (Figure 4, right). Thus, a direct transfer was not observed with the kinetochore particles even with Dam1‐4D, in contrast to Ndc80C‐nanobeads. We speculate that the different behavior of an Ndc80C‐nanobead and a purified kinetochore particle with Dam1‐4D might be due to different distributions and orientations of the Ndc80Cs, that is, Ndc80Cs might be randomly distributed around the nanobead (diameter ≈100 nm) and orient all directions (Figure 4, left), whereas Ndc80Cs on the kinetochore particle may have a small footprint and orient mostly in one direction, i.e. toward a MT^[^ ^50^, ^51^ ^]^ (Figure 4, right).

If native kinetochores do not show direct transfer between MTs during error correction, the disruption of end‐on attachment may precede formation of lateral attachment to another MT, as shown in Figure 3 (step 1, 2 followed by either 3 or 4). However, Dam1‐4D rarely led to detachment of either Ndc80C‐nanobeads or purified kinetochore particles from a MT end in the absence of a crossing MT, even if the end‐on attachment was weakened by Dam1‐4D.^[^ ^41^ ^]^ In addition, Dam1‐4D led to only slow kinetochore detachment from the MT end (over 30 min or longer) in cells.^[26]^ How can we explain these observations? We speculate that a rapid disruption of the end‐on attachment may occur only in the context of aberrant kinetochore‐MT interactions – more specifically, syntelic attachment (Figure 3, step 1). For example, if two MTs from the same pole attach to sister kinetochores (Figure 3, step 1), even slight difference in their dynamics (growth speed, timing of rescue and catastrophe, etc.) would generate a shearing force between sister kinetochores.^[^ ^16^ ^]^ This would cause disruption of already weakened end‐on attachment of one sister kinetochore. Once this happens, the shearing force should be released and the end‐on attachment of the other sister kinetochore should remain for the time being, even if it is weakened (Figure 3, step 2). Such a mechanism would prevent both sister kinetochores simultaneously losing MT attachment and contribute to avoiding a chromosome drifting away from the spindle during error correction. If a chromosome drifts away, it would have to be caught again on a MT extending from a spindle pole, which is time consuming,^[^ ^11^ ^]^ thus delaying establishment of biorientation.

## HOW THE SWAP OF KINETOCHORE–MT INTERACTIONS STOPS UPON BI‐ORIENTATION

An aberrant kinetochore–MT interaction is resolved through the exchange (swap) of kinetochore–MT interaction (Figure 1, step 4 and 5). However, once biorientation is formed (Figure 1, step 6), the kinetochore–MT interaction must be stabilized so that the exchange of kinetochore–MT interaction stops. It has been widely accepted that the stability of kinetochore–MT interaction is regulated by tension applied on this interaction^[^ ^17^, ^42^ ^]^: with aberrant kinetochore–MT interaction, tension is weak and kinetochore–MT interaction is destabilized. When biorientation is established, sister kinetochores are pulled in opposite directions and tension is applied across sister kinetochores, which stabilizes kinetochore–MT interaction. However, it is still not completely understood how tension stabilizes kinetochore–MT interaction. To explain this, several models have been proposed as below.

### Aurora B spatial separation model

**FIGURE 5:**
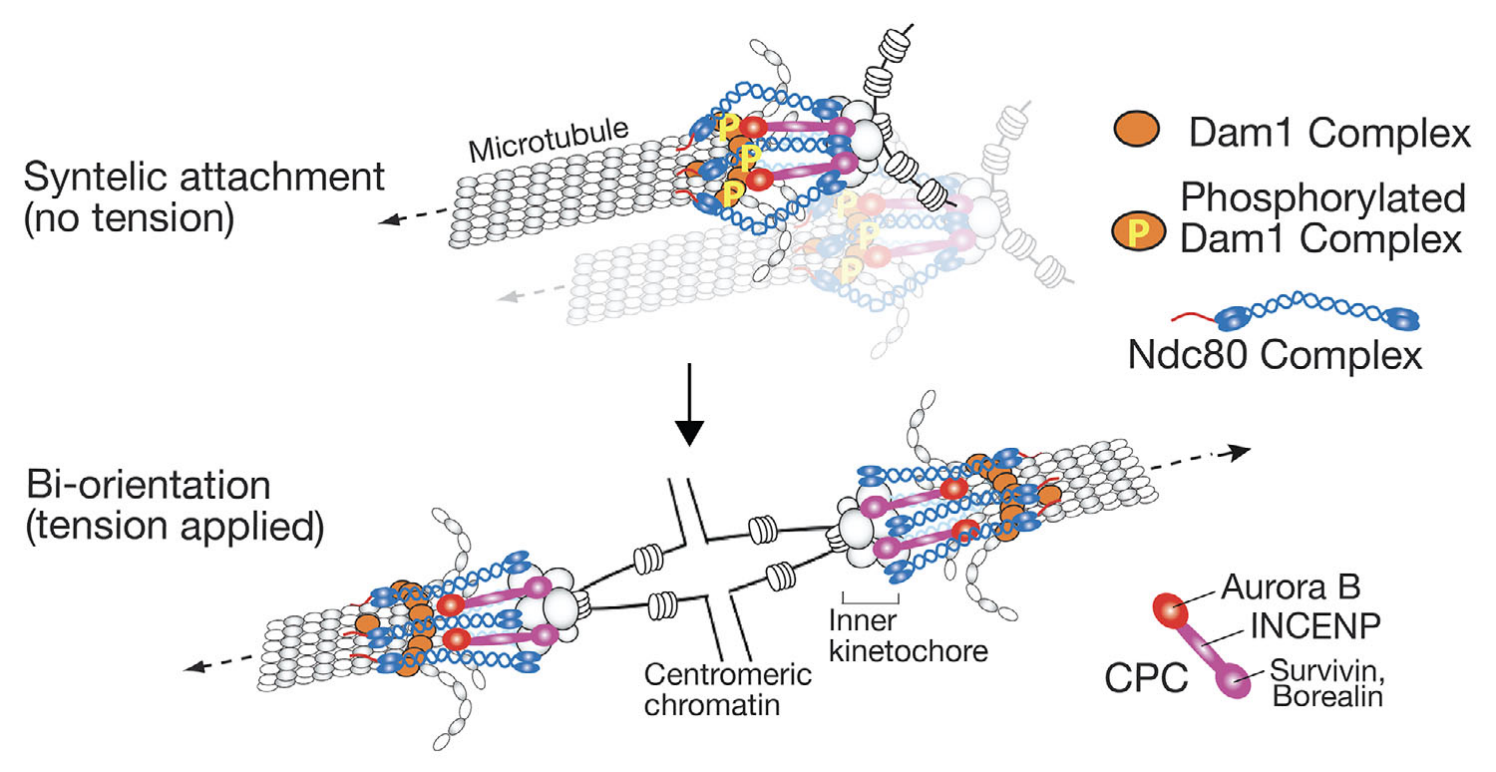
Aurora B spatial separation model explains how tension, applied across sister kinetochores, stabilizes kinetochore–MT interactions.^[^ ^5^ ^]^ Aurora B is recruited to the inner kinetochore and centromeric nucleosome by INCENP and other CPC components. During syntelic attachment (aberrant kinetochore–MT interactions), Aurora B can reach Dam1C and phosphorylates its components, which destabilized kinetochore attachment to the MT end (top). When biorientation is established, Ndc80Cs are stretched and Aurora B cannot reach Dam1C, which stabilizes kinetochore–MT interactions (bottom). Note that only the CPCs at the inner kinetochore are shown in diagram for simplicity, but CPCs at the centromeric nucleosome (not shown here) should also behave similarly

As discussed in the previous section, Aurora B kinase plays a central role in destabilizing aberrant kinetochore–MT interaction. However, when biorientation is established and tension is applied, this Aurora B function needs to cease or to be overcome – otherwise kinetochore–MT interaction would not be stabilized. To consider this mechanism, we should first understand where Aurora B localizes. Several studies have established that the CPC (containing Aurora B) is recruited to the centromere. This recruitment relies on phosphorylation of histone H3 and H2A by Haspin and Bub1 kinases, respectively.^[^ ^52^, ^53^, ^54^, ^55^ ^]^ H3 phosphorylation directly recruits Survivin, while H2A phosphorylation recruits Shugoshin which in turn binds Survivin or Borealin.^[^ ^52^, ^53^, ^54^, ^55^, ^56^ ^]^ Given the CPC localization at the centromere, it can be explained how Aurora B function stops promoting error correction in a tension dependent manner, as follows: When tension is applied, the kinetochore is stretched, leading to spatial separation between Aurora B (localizing at the centromere/inner kinetochore) and its outer kinetochore substrates (Ndc80 N‐terminus and Dam1C components), whose phosphorylation is important for error correction as discussed in INTRODUCTION (Figure 5). This spatial separation results in dephosphorylation of the outer kinetochore substrates, which stabilizes the kinetochore attachment to the MT end (Aurora B spatial separation model).^[^ ^33^, ^57^ ^]^

Though INCENP is an elongated protein, Ndc80C contains a long coiled‐coil region that exceeds INCENP in length when stretched. This should allow the spatial separation between Aurora B and its outer kinetochore substrates (Dam1C and Ndc80 N‐terminus) when tension is applied^[^ ^58^, ^59^ ^]^ (Figure 5, bottom). On the other hand, Ndc80C has a kink in the middle and, when tension is not applied, it can bend flexibly at the kink,^[^ ^60^, ^61^, ^62^ ^]^ which would allow access of Aurora B to its outer kinetochore substrates (Figure 5, top). This access of Aurora B may require a certain length of INCENP – in fact, 209 amino‐acid deletion in middle of human INCENP was able to promote biorientation, but 327 amino‐acid deletion in middle of yeast INCENP showed defects in biorientation.^[^ ^63^, ^64^ ^]^ The Aurora B spatial separation model is supported by a series of evidence in budding yeast and mammalian cells: First, phosphorylation of outer kinetochore components by Aurora B is reduced in a tension‐dependent manner.^[^ ^40^, ^57^, ^65^ ^]^ Second, ectopic targeting of Aurora B (with its activator INCENP) to the outer kinetochore destabilizes kinetochore–MT interaction during metaphase in budding yeast (Garcia‐Rodriguez and Tanaka, unpublished) and in human cells.^[^ ^66^ ^]^ Furthermore, the Aurora B spatial separation model explains why the CPC relocalizes from the centromere/inner kinetochore to the spindle midzone at the anaphase onset.^[^ ^34^ ^]^ If Aurora B were to remain at the centromere/inner kinetochore after tension is reduced (due to loss of sister chromatid cohesion) during anaphase, it would destabilize kinetochore–MT interaction. Indeed, this was experimentally demonstrated using INCENP (and other) mutants that retain Aurora B at kinetochores during anaphase.^[^ ^67^, ^68^, ^69^ ^]^

The Aurora B spatial separation model predicts that Aurora B localization at (or near) centromeres are required for error correction and biorientation. However, this notion was challenged by the finding that Survivin, which recruits Aurora B–INCENP to centromeres (centromere nucleosomes),^[^ ^34^, ^63^ ^]^ can become dispensable for biorientation in budding yeast.^[^ ^70^ ^]^ This raised the possibility that Aurora B localization at centromeres is not required for bi‐orientation – if so, the spatial separation model would be excluded, as this model relies on Aurora B at (or near) centromeres being crucial for biorientation. However, an alternative possibility is that a Survivin‐independent mechanism recruits Aurora B–INCENP to (or near) centromeres. Indeed, this alternative possibility has proved to be the case. It has been found that, independently of Survivin, INCENP directly interacts with the Mcm21–Ctf19 subcomplex (CENP‐O–CENP‐P subcomplex in mammalian cells) at the inner kinetochore to recruit Aurora B in budding yeast.^[^ ^35^, ^36^ ^]^ As Mcm21–Ctf19 is relatively close to the centromere (<10–20 nm) and far away (> 60 nm) from the Aurora B substrates (Dam1C and Ndc80 N‐terminus) during end‐on MT attachment,^[^ ^51^ ^]^ the spatial separation should still occur between Aurora B, recruited by Mcm21‐Ctf19, and its outer kinetochore substrates, when biorientation is established. In mammalian cells, although CENP‐O/P may not be sufficient for Aurora B recruitment,^[^ ^71^ ^]^ Aurora B is at least recruited both to centromeres and to (or near to) the inner kinetochore.^[^ ^37^, ^72^ ^]^

The Survivin‐dependent centromere recruitment and Survivin‐independent inner kinetochore recruitment work redundantly for Aurora B–INCENP, though the former is usually predominant.^[^ ^35^, ^36^ ^]^ If both Aurora B recruitment mechanisms are defective, most sister kinetochore pairs show defects in biorientation. However, if INCENP is artificially recruited to another inner kinetochore component in this situation, biorientation is restored in majority of sister kinetochore pairs in budding yeast.^[^ ^35^, ^36^ ^]^ Thus, the localization of Aurora B–INCENP at centromeres or inner kinetochores is essential for error correction and biorientation, which supports the Aurora B spatial separation model.

### Other models explaining tension‐dependent regulation of kinetochore–MT interactions

Although the Aurora B spatial separation model is supported by several pieces of evidence, it is very possible that other mechanisms are additionally required to ensure tension‐dependent stabilization of kinetochore–MT interactions when biorientation is established. Such mechanisms may involve dynamic regulation of Aurora B kinase (or the CPC including Aurora B). Proposed mechanisms are as follows: First, Aurora B may be released from its localization sites and reach the Aurora B kinase substrates (Dam1C and Ndc80 N‐terminus).^[^ ^73^ ^]^ If the localization sites are close to the substrates, Aurora B would more frequently reach substrates. Thus, this is consistent with the Aurora B separation model, but it helps Aurora B to reach its substrates located at larger distance. Second, a small pool of Aurora B may be recruited to the outer kinetochore, close to the kinetochore–MT interface, in early mitosis.^[^ ^37^, ^74^ ^]^ It is proposed that localization of Aurora B at the outer kinetochore may be removed by tension, leading to stabilization of kinetochore–MT interaction when tension is applied with biorientation.^[^ ^75^ ^]^ Third, INCENP and Borealin (both are components of CPC) have MT‐binding domains^[^ ^59^, ^76^, ^77^, ^78^ ^]^ and it is proposed that MT binding of the CPC may regulate phosphorylation of Aurora B substrates (at outer kinetochores) in a tension dependent manner.^[^ ^79^ ^]^ Fourth, tension may cause stretching of CPC components, which could reduce the activity of Aurora B. In particular, it is proposed that INCENP may have a spring‐like function, to regulate Aurora B activity in a tension‐dependent manner.^[^ ^59^, ^63^ ^]^ Fifth, Borealin undergoes phase separation at least in vitro^[^ ^80^ ^]^ and it is proposed that phase separation facilitates accumulation of the CPC specifically when aberrant kinetochore–MT interactions are present.^[^ ^81^ ^]^

Alternatively, factors other than Aurora B may be involved in tension‐dependent regulation of kinetochore–MT interactions. When biorientation is established, such factors may overcome the function of Aurora B, thus stabilizing kinetochore–MT interactions. Proposed such factors are as follows: First, when tension is applied, the rate of conversion from MT polymerization to depolymerization (catastrophe) decreases in vitro, independently of Aurora B. This in turn reduces the rate of kinetochore detachment from a MT.^[^ ^49^ ^]^ Second, although Stu2 (and its human orthologue ch‐TOG) is known as a MT polymerase, the fraction of Stu2 at the kinetochore may have dual roles in stabilizing and destabilizing kinetochore–MT interactions with high and low tension, respectively, independently of MT dynamics and Aurora B kinase.^[^ ^82^, ^83^, ^84^ ^]^ Third, PP1 and PP2A phosphatases are recruited to the kinetochore by KNL1 (Spc105 in budding yeast) and other factors to regulate spindle‐assembly checkpoint.^[^ ^85^, ^86^, ^87^, ^88^ ^]^ It is also implicated that PP1 and PP2A regulate kinetochore–MT interactions^[^ ^89^, ^90^, ^91^, ^92^ ^]^ and this may happen in a tension‐dependent manner.^[^ ^93^, ^94^ ^]^ For example, it is possible that tension causes the structural change of Aurora B targets, which may allow phosphatases to dephosphorylate them more efficiently. Fourth, in addition to Aurora B kinase, Mps1 kinase is required to resolve aberrant kinetochore–MT interactions from yeast to human cells.^[^ ^95^, ^96^, ^97^, ^98^, ^99^ ^]^ Mps1 kinase is recruited to the kinetochore by the Ndc80C^[^ ^100^, ^101^, ^102^ ^]^ and phosphorylates outer kinetochore components Ndc80, Spc105, and Ska3 to promote biorientation.^[^ ^103^, ^104^, ^105^ ^]^ The Mps1 activity at kinetochores is highest without MT attachment and reduced once biorientation is established.^[^ ^100^, ^101^, ^106^ ^]^ Fifth, Aurora A around spindle poles facilitates Ndc80 phosphorylation, when chromosomes oscillate on the metaphase spindle, which resolve any remaining aberrant kinetochore–MT interactions.^[^ ^107^, ^108^, ^109^ ^]^ When biorientation becomes mature (i.e., a larger number of MTs attach to one kinetochore in mammalian cells), chromosome oscillation may be reduced, which could stabilize kinetochore–MT interactions.

## FUTURE PERSPECTIVE

We have discussed two fundamental mechanisms for establishing chromosome biorientation, that is, how kinetochore–MT interactions are exchanged (swapped) during error correction and how tension stops this exchange and stabilizes kinetochore–MT interactions when biorientation is established. For swapping kinetochore–MT interactions, evidence suggests that differential regulation of end‐on attachment and lateral attachment by Aurora B is a key mechanism to promote this swapping.^[^ ^26^, ^41^ ^]^ However, the role of other regulators of kinetochore–MT interactions in this process is still unclear. For example, Stu2‐dependent MT generation at kinetochores^[^ ^8^, ^11^ ^]^ and Kar3‐ (or Dynein‐) dependent kinetochore sliding along a MT^[^ ^6^, ^110^ ^]^ may be involved in swapping kinetochore–MT interactions. Moreover, other regulators (or regulations) for biorientation, for example, Stu2‐dependent stabilization of kinetochore–MT interactions^[^ ^82^, ^83^ ^]^ and Mps1‐dependent error correction^[^ ^96^, ^98^ ^]^ may be involved. To address which steps of kinetochore–MT swapping (e.g., disruption of end‐on attachment) are regulated by these factors, new methods for analyzing this process will be instrumental. For example, if kinetochore–MT swapping can be directly visualized in vivo or reconstituted in vitro, it would greatly facilitate new discoveries.

For stopping the exchange of kinetochore–MT interactions, a series of evidence supports the Aurora B spatial separation model. However, it is plausible that additional mechanisms are required to ensure this process. Indeed, several additional mechanisms are proposed – some mechanisms involve dynamic regulation of Aurora B kinase and other mechanisms rely on regulators other than Aurora B. It will be important to evaluate which mechanisms are more directly involved in stopping the exchange of kinetochore–MT interactions in a tension dependent manner. It is also crucial to determine which mechanisms are essential for this process while others are required only for fine tuning of this process. However, it is not easy to answer these questions, as some of the proposed mechanisms would work redundantly with others while some others are involved in multiple regulations. We will need to disentangle such complications to address the above questions. Development of new techniques and/or combination of existing techniques will help us make new discoveries. Chromosome biorientation is at the heart of mechanisms ensuring genetic integrity in proliferating cells. Failure in establishing chromosome biorientation is a major etiology of human diseases characterized by chromosome instability and aneuploidy.^[^ ^111^, ^112^, ^113^ ^]^ Understanding mechanisms of error correction leading to chromosome biorientation should provide new insight into the cause of these diseases.

## CONFLICT OF INTEREST

The authors declare no competing financial interests.

## Data Availability

Data sharing is not applicable to this article as no new data were created or analyzed in this study.
